# Advanced Wastewater Treatment Engineering—Investigating Membrane Fouling in both Rotational and Static Membrane Bioreactor Systems Using Empirical Modelling

**DOI:** 10.3390/ijerph13010100

**Published:** 2016-01-05

**Authors:** Parneet Paul, Franck Anderson Jones

**Affiliations:** 1 Department of Civil Engineering, School of Natural and Built Environments, Faculty of Science, Engineering and Computing, Kingston University, Penrhyn Road, Kingston upon Thames, Surrey KT1 2EE, UK; 2 Department of Mechanical, Aerospace and Civil Engineering, College of Engineering, Design and Physical Sciences, Brunel University, Uxbridge, Middlesex UB8 3PH, UK; mepgfaj@brunel.ac.uk

**Keywords:** wastewater, membrane bioreactor (MBR), rotating membranes, fouling, modelling, cake filtration

## Abstract

Advanced wastewater treatment using membranes are popular environmental system processes since they allow reuse and recycling. However, fouling is a key limiting factor and so proprietary systems such as Avanti’s RPU-185 Flexidisks membrane bioreactor (MBR) use novel rotating membranes to assist in ameliorating it. In earlier research, this rotating process was studied by creating a simulation model based on first principles and traditional fouling mechanisms. In order to directly compare the potential benefits of this rotational system, this follow-up study was carried out using Avanti’s newly developed static (non-rotating) Flexidisks MBR system. The results from operating the static pilot unit were simulated and modelled using the rotational fouling model developed earlier however with rotational switching functions turned off and rotational parameters set to a static mode. The study concluded that a rotating MBR system could increase flux throughput when compared against a similar static system. It is thought that although the slowly rotating spindle induces a weak crossflow shear, it is still able to even out cake build up across the membrane surface, thus reducing the likelihood of localised critical flux being exceeded at the micro level and lessening the potential of rapid trans-membrane pressure increases at the macro level.

## 1. Introduction

In the wastewater reuse, recycling and reclamation sectors, considerable research is on-going to create environmental systems engineering processes that address the water scarcity and security issues facing large parts of the world. As part of this work advanced wastewater treatment systems using membrane processes are becoming very popular as alternatives to traditional processes since they allow wastewater reuse and recycling. Thus, static or non-rotating membrane bioreactors (MBRs) are coveted systems widely used in filtration technologies for biomass separation and to remove microorganisms, particles and turbidity [1,2]. This is mainly because they offer many beneficial advantages over conventional treatment processes. These advantages include reduced footprint and sludge production by achieving high biomass concentrations in the bioreactor; high quality of reusable effluent produced as permeate; and, high nitrification and particles’ removal rates [3,4]. However well these static MBR systems perform they might be superseded in the future by newly developed rotating MBR systems that potentially have reduced energy consumption and fouling [5]. Rotating MBR systems typically induce high shear effects on the membrane surface thereby reducing associated fouling whilst minimising energy usage. These systems have been shown to yield high permeate flux in the ultra-filtration (UF) range [6], whilst the very high shear rate simultaneously yields a good system performance by preventing cake formation and subsequent increased concentration polarisation [7]. For instance in Jørgensen *et al*. [8] study that used rotating ceramic membrane discs fouled by sludge, the shear directly impacted on the fouling levels, and in consequence a model was developed that linked the shear rate to the limiting flux.

A major defining issue in the application of MBR systems for water processes is the phenomenon known as fouling, which in turn reduces productivity and increases operational and maintenance costs [4,9]. Fouling by non-Newtonian fluids such as activated sludge used in MBR systems is a key limiting factor in UF membrane processes. The true origin of fouling has yet to be fully defined, although many researchers widely acknowledge that SMP (soluble microbial products) and EPS (extracellular polymeric substances) are the most likely fouling agents [9]. This is since the build-up of SMP and EPS can cause reduction in membrane permeability [10,11]. Additional factors affecting fouling mechanisms include: scaling; biofilm formation; operating conditions, such as pH; temperature and flow rates; and, solution properties such as particle size distribution [9]. According to Hermia [12], during constant pressure UF, three major fouling mechanisms are likely to occur, and these are generally known as pore constriction; pore blocking (typically divided into either complete or intermediate); and cake filtration. The aforementioned fouling mechanisms describe the accumulation of particles, solutes, and colloids inside the membrane’s pores and on the membrane’s surface leading to a reduction in the diameter of open pores (*i.e.*, pore constriction); an obstruction of the pores by particles larger than the membrane’s pore size (*i.e*., pore blockage) and the deposition of layers of particles onto the blocked membrane’s surface (*i.e.*, cake filtration). Additionally, depending on the composition of the fluid being filtered and the interactions between the membrane and the bulk liquid, one fouling process may dominate over the other two or conversely all three fouling mechanisms may occur simultaneously during the filtration time [12].

Many fouling studies have been carried out to date using pilot units in order to determine the best operating conditions of MBR systems, although currently, due to the complexity of the biomass matrix which includes living microorganisms, no definitive theories on membrane fouling have been established [3]. Different approaches have also been developed for modelling the physical and biological aspects of membrane fouling in a normal non-rotational MBR system. For instance Meng *et al*. [13] established a fractal permeation model while Liu *et al*. [14] presented an empirical hydrodynamic model. Duclos-Orsello *et al*. [15] introduced a fouling model that combined all three classical fouling mechanisms [12], which was later used by Paul [16] as a starting point for a greatly refined model for a side-stream MBR that incorporated both hydrodynamics and SMP effects.

Notwithstanding numerous experimental studies, simulation and modelling of rotating MBR systems is still a nascent topic to date due to the poor understanding and great complexity of the system hydrodynamics involved. The dearth of actual commercially available systems on the market that could be studied does not help the situation either. Since mathematical modelling can be used to simulate flux decline and thus potentially afford a greater understanding of the membrane fouling mechanisms involved, the aims of this research work were to create:

- A fully comprehensive fouling model for a static MBR system incorporating all three classical fouling mechanisms.
- A complete fouling model integrating the three conventional fouling processes for a rotating MBR system that would also include the rotational hydrodynamics effect.
- A comparison of both MBR systems and identification of the impact of the rotational effect on the rotating MBR system.

Full data sets would be used to validate both models under short and medium term filtration conditions.

## 2. Theoretical Approach

The fouling model for the rotating MBR system used in this study was developed earlier by Paul and Jones [17] and extensively tested for validity. These equations will once again be reiterated in this section.

Using the power law for non-Newtonian fluids, such as activated sludge, the viscosity (Pa·s) of mixed liquor suspended solids (MLSS) in a MBR is proportional to the shear rate as depicted in Equation (1) [5].

(1) $$\mu =m{\dot{\gamma }}^{n-1}$$

To calculate the shear rate over the rotating membrane, consideration must be given to the type flow through the membrane module. The shear rate based on different flow regimes is computed using Equation (2) [18].

(2) if,{ReNN≤2.105, laminar flow, γ˙=1.81.(kω.ω)1.5.ro.ν−0.5ReNN≥2.105, turbulent flow, γ˙=0.057.(kω.ω)1.8.ro1.6.ν−0.8

**Figure 1:**
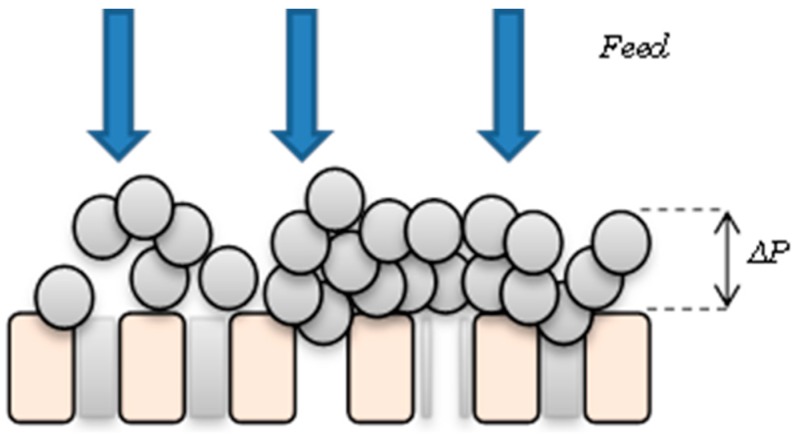
Diagram of the combined fouling mechanisms. Colloids or small particles constrict the pores while larger particles blocked them, forming a cake.

Firstly, it was assumed that the pores were cylindrical and uniformly distributed throughout the membrane, so that fluid flow could be described by Hagen-Poiseuille flow. Hence, pore constriction occurs through all open pores, and gradually the membrane surface becomes obstructed by aggregates forming a somewhat uneven blocked area. Once the pores are blocked by aggregates pore constriction is stopped. Consequently, a cake layer will form over any blocked area. The resistance of this deposit layer is time dependent with regions of greatest resistance delivering the smallest flux. However, in reality the actual membrane fouling process is extremely complex in nature with usually all effects occurring simultaneously. Nevertheless, to simplify the model the above assumptions are made as well as overlooking the effect of frictional forces and temperature. Figure 1 shows the combined fouling mechanisms in the model.

In a similar manner to the reformulation of the Duclos-Orsello *et al.* [15] model undertaken by Paul [16], the bulk concentration *C_b_* (g/L) is replaced by the MLSS concentration, *C_MLSS_* (g/L). 

Assuming the membrane rotates around a fixed axis (here defined as an imaginary straight line passing through the shaft) with angular velocity, ω, and using the pore constriction model, the unblocked flux, *J_u_* (m·s^−1^), is defined as a function of time within the unblocked area, $A_{u}$ (m^2^), as shown in Equation (3) [15,16].

(3) $$\frac{J_{u}(t)}{J_{0}}=\frac{1}{{\left(1+\beta .Q_{0}.C_{\mathrm{MLSS}}.t\right)}^{2}};\mathrm{where}\beta =\frac{\sigma_{a}}{\pi .{\left(r_{p}\right)}^{2}.\delta_{m}}\;\to \;J_{u}\left(t\right)=\frac{r_{0}^{\prime }.k_{\omega }.\omega }{{\left(1+\beta .Q_{0}.C_{\mathrm{MLSS}}.t\right)}^{2}};\mathrm{where}J_{0}=r_{0}^{\prime }.k_{\omega }.\omega$$

As the membrane fouls with time, the unblocked area also decreases at the same rate, and the rate of unblocked area reduction is given in Equation (4).

(4) $$\frac{{\mathrm{dA}}_{u}}{A_{u}}=\frac{-\alpha .C_{\mathrm{MLSS}}.r_{0}^{\prime }.k_{\omega }.\omega }{{\left(1+\beta .Q_{0}.C_{\mathrm{MLSS}}.t\right)}^{2}}.\mathrm{dt}$$

Assuming that at time *t* = 0, the initial unblocked area through the membrane is *A_u_*_0_ (m^2^), then by integrating Equation (4) between the time filtration boundaries, Equation (5) is derived.

(5) $$A_{u}(t)=A_{u_{0}}.e^{\frac{\alpha .r_{0}^{\prime }.k_{\omega }.\omega }{\beta .Q_{0}}.\left(\frac{1}{1+\beta .Q_{0}.C_{\mathrm{MLSS}}.t}-1\right)}$$

By combining Equations (3) and (5), the volumetric flow rate (*Q_u_*, m^3^/s) through open pores can be calculated as shown in Equation (6).

(6) $$Q_{u}\left(t\right)=A_{u}\left(t\right).J_{u}\left(t\right)=\frac{A_{u_{0}}.r_{0}^{\prime }.k_{\omega }.\omega }{{\left(1+\beta .Q_{0}.C_{\mathrm{MLSS}}.t\right)}^{2}}.e^{\left\{\frac{\alpha .r_{0}^{\prime }.k_{\omega }.\omega }{\beta .Q_{0}}.\left(\frac{1}{1+\beta .Q_{0}.C_{\mathrm{MLSS}}.t}-1\right)\right\}}$$

The blocked flux, *J_b_* (m·s^−1^), can be calculated from Equation (7) using Darcy’s Law and a resistance in-series approach, whilst the trans-membrane pressure (TMP), can be expressed in terms of density and angular velocity in Equation (8) [18].

(7) $$J_{b}=\frac{\mathrm{TMP}}{\mu .\left(R_{\mathrm{in},b}+R_{b}\right)}$$

(8) $$\mathrm{TMP}=-\mathrm{PT}-\left(\frac{1}{4}.\rho_{f}.{\left(k_{\omega }.\omega .r_{o}\right)}^{2}\right)$$

Once the pore constriction stops at time, *t_b_*, the time at which a pore was first blocked, *R_in,b_* can be calculated from Equation (9) [15].

(9) $$R_{\mathrm{in},b}=R_{m}{\left(1+\beta .Q_{0}.C_{\mathrm{MLSS}}.t_{b}\right)}^{2}$$

The resistance of the particles deposited increases with time due to the growth in mass (or thickness) of the cake layer, and within the cake filtration model, the resistance *R_b_* is determined in Equation (10).

(10) $$\frac{{\mathrm{dR}}_{b}}{\mathrm{dt}}=f^{\prime }.R^{\prime }.J_{b}.C_{\mathrm{MLSS}}$$

Assuming, no loss in area, the blocked area, *A_b_* (m^2^), is given by Equation (11), and is directly proportional to the unblocked area *A_u_* (m^2^) at time *t_b_*.

(11) $$\frac{{\mathrm{dA}}_{b}}{{\mathrm{dt}}_{b}}=-\frac{{\mathrm{dA}}_{u}}{{\mathrm{dt}}_{b}}\to A_{b}\left(t_{b}\right)=\overset{t}{\underset{0}{\int }}\left(\frac{A_{u0}.\alpha .C_{\mathrm{MLSS}}.{r\prime }_{0}.k_{\omega }.\omega }{{\left(1+\beta .Q_{0}.C_{\mathrm{MLSS}}.t_{b}\right)}^{2}}.e^{\left\{\frac{\alpha .{r\prime }_{0}.k_{\omega }.\omega }{\beta .Q_{0}}.\left(\frac{1}{1+\beta .Q_{0}.C_{\mathrm{MLSS}}.t_{b}}-1\right)\right\}}\right){\mathrm{dt}}_{b}$$

At low rotational speeds, the flow is considered laminar and by combining Equations (1), (2), (7)–(9) and (11), the volumetric flow rate (*Q_b_*, m^3^/s) through the blocked pores is given by Equation (12).

(12) $$Q_{b}(t)=\frac{-\mathrm{PT}-\left(\frac{1}{4}.\rho_{f}.{\left(k_{\omega }.\omega .r_{o}\right)}^{2}\right)}{m.{\left(1.81.{\left(k_{\omega }.\omega \right)}^{1.5}.r_{o}.\nu^{-0.5}\right)}^{n-1}.\left(R_{m}{\left(1+\beta .Q_{0}.C_{\mathrm{MLSS}}.t_{b}\right)}^{2}+R_{b}\right)}.\overset{t}{\underset{0}{\int }}\left(\frac{A_{u0}.\alpha .C_{\mathrm{MLSS}}.{r\prime }_{0}.k_{\omega }.\omega }{{\left(1+\beta .Q_{0}.C_{\mathrm{MLSS}}.t_{b}\right)}^{2}}.e^{\left\{\frac{\alpha .{r\prime }_{0}.k_{\omega }.\omega }{\beta .Q_{0}}.\left(\frac{1}{1+\beta .Q_{0}.C_{\mathrm{MLSS}}.t_{b}}-1\right)\right\}}\right){\mathrm{dt}}_{b}$$

Hence the total volumetric flow rate through the membrane is expressed as the summation of the flow rate through the unblocked *Q_u_* and blocked *Q_b_* pores respectively as shown in Equation (13).

(13) $$\begin{array}{l} Q\left(t\right)\\ =\frac{A_{u_{0}}.{r\prime }_{0}.k_{\omega }.\omega }{{\left(1+\beta .Q_{0}.C_{\mathrm{MLSS}}.t\right)}^{2}}.e^{\left\{\frac{\alpha .{r\prime }_{0}.k_{\omega }.\omega }{\beta .Q_{0}}.\left(\frac{1}{1+\beta .Q_{0}.C_{\mathrm{MLSS}}.t}-1\right)\right\}}\\ +\frac{-\mathrm{PT}-\left(\frac{1}{4}.\rho_{f}.{\left(k_{\omega }.\omega .r_{o}\right)}^{2}\right)}{m.{\left(1.81.{\left(k_{\omega }.\omega \right)}^{1.5}.r_{o}.\nu^{-0.5}\right)}^{n-1}.\left(R_{m}{\left(1+\beta .Q_{0}.C_{\mathrm{MLSS}}.t_{b}\right)}^{2}+R_{b}\right)}.\overset{t}{\underset{0}{\int }}\left(\frac{A_{u0}.\alpha .C_{\mathrm{MLSS}}.{r\prime }_{0}.k_{\omega }.\omega }{{\left(1+\beta .Q_{0}.C_{\mathrm{MLSS}}.t_{b}\right)}^{2}}.e^{\left\{\frac{\alpha .{r\prime }_{0}.k_{\omega }.\omega }{\beta .Q_{0}}.\left(\frac{1}{1+\beta .Q_{0}.C_{\mathrm{MLSS}}.t_{b}}-1\right)\right\}}\right){\mathrm{dt}}_{b} \end{array}$$

It is interesting to note that, at *t* = 0, *t_b_* = 0, thus, *Q* = *Q*_0_.

When describing the hydrodynamic regime in this model, the air scouring flux, *J_air_* (m/s), is a key parameter for the management and prevention of membrane fouling in most submerged MBR systems. As such, cake layer growth rate depends on the scouring energy induced by the aeration. Furthermore, rotation in rotating MBRs produces a torque that induces additional shear effects to reduce fouling on the membrane surface. Rightfully so, since the rotating MBR has a very low rotational speed of 2.09 rad/s (or 20 revolutions per minute), the above-mentioned scenario and ensuing equations will be correct. However, it is worth mentioning that at very high rotational speeds there is a high possibility that the air scouring effect will be significantly less than that induced by rotation. The net total effect responsible for reducing fouling on the membrane can tentatively be calculated by the summation of the air scouring and rotational effects. However in hindsight, at some point during the filtration process, these two effects work in opposing directions. This fact alone ultimately poses a physical limitation to the model since a completely isolated hydrodynamic study of the shear stresses will be required, which is not the scope of this study. The cake’s resistance is consequently decreased to allow the system to gain flux due to these membrane cleaning effects. To account for these changes, an additional removal term was added to the rate of membrane blocked area, and was defined as the flux induced by the air scouring flow combined with rotational effects. This additional removal term is also in-line with Liang *et al.* [19] cake’s formulation equation that accounted for the change in reversible fouling due to cake build-up. An analogous reformulation is found in Equation (14) but includes air scouring and rotational effects.

(14) $$\frac{{\mathrm{dR}}_{b}}{\mathrm{dt}}=f^{\prime }.R^{\prime }.J_{b}.C_{\mathrm{MLSS}}-g_{o}.\left(\alpha_{v}.J_{\mathrm{air}}-k_{\omega }.\omega .r_{o}\right).\delta .R_{c}$$

Subsequently, the blocked area, *A_b_* (m^2^), is mathematically given by Equation (15).

(15) $$\;\;\;\frac{{\mathrm{dA}}_{b}}{\mathrm{dt}}=\alpha .J_{u}.A_{u}.C_{\mathrm{MLSS}}-k_{A_{b}}.\left(\alpha_{v}.J_{\mathrm{air}}-k_{\omega }.\omega .r_{o}\right).\theta_{c}(t)$$

### 2.1. Static Square-Shaped MBR System

Equation (13) can be reduced to Equation (16), if the rotational switching function is removed so that the model reverts to a submerged static MBR system that now simply includes the air scouring term.

(16) $$\begin{array}{c} Q(t)=\frac{A_{u_{0}}.J_{0}}{{\left(1+\beta .Q_{0}.C_{\mathrm{MLSS}}.t\right)}^{2}}.e^{\left\{\frac{\alpha .J_{0}}{\beta .Q_{0}}.\left(\frac{1}{1+\beta .Q_{0}.C_{\mathrm{MLSS}}.t}-1\right)\right\}}\\ +\frac{-\mathrm{PT}}{\mu .\left(R_{m}{\left(1+\beta .Q_{0}.C_{\mathrm{MLSS}}.t_{b}\right)}^{2}+R_{b}\right)}.\overset{t}{\underset{0}{\int }}\left(\frac{A_{u0}.\alpha .C_{\mathrm{MLSS}}.J_{0}}{{\left(1+\beta .Q_{0}.C_{\mathrm{MLSS}}.t_{b}\right)}^{2}}.e^{\left\{\frac{\alpha .J_{0}}{\beta .Q_{0}}.\left(\frac{1}{1+\beta .Q_{0}.C_{\mathrm{MLSS}}.t_{b}}-1\right)\right\}}\right){\mathrm{dt}}_{b} \end{array}$$

The only prevalent hydrodynamic factor to take into account during operation of the static square-shaped MBR is the coarse bubble air scour that is mainly used to mitigate cake growth and thus hamper fouling. Consequently, the air scouring flux *J_air_* (m·s^−1^) also becomes a vital parameter for the management of fouling in this static square-shaped MBR system. Hence, this air scour removal term was added to the rate of fouling resistance build up and rate of increase in blocked area. This is a comparable formulation that is also in-line with Liang *et al*. [19] rate of membrane biomass buildup equation.

In a similar manner for the rotational MBR model in Equations (14) and (15), the hydrodynamics effect in the form of air scour alone can be reduced simply to Equations (17) and (18).

(17) $$\frac{{\mathrm{dR}}_{b}}{\mathrm{dt}}=f^{\prime }.R^{\prime }.J_{b}.C_{\mathrm{MLSS}}-g_{o}.\left(\alpha_{v}.J_{\mathrm{air}}\right).\delta .R_{c}$$

(18) $$\;\;\frac{{\mathrm{dA}}_{b}}{\mathrm{dt}}=\alpha .J_{u}.A_{u}.C_{\mathrm{MLSS}}-k_{A_{b}}.\left(\alpha_{v}.J_{\mathrm{air}}\right).\theta_{c}(t)$$

## 3. Materials and Method

### 3.1. Materials

**Figure 2:**
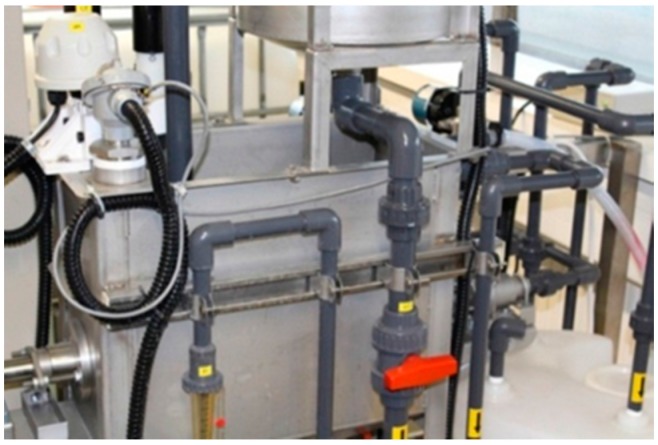
Rotating membrane bioreactor (MBR) system RPU-185 in operation. The membrane module is located in the batch tank for filtration purposes.

The rotating MBR pilot unit used to generate short and medium term filtration data for testing of the fouling model consisted of an UF module of 36 circular flat membrane sheets as shown in Figure 2 (FUV-185-A15R Flexidisks by Avanti Membrane Technology, Walnut, CA, USA). These circular membrane sheets were attached to a single shaft rotating via an electrical motor with an operational speed of 20 revolutions per minute (RPM). Each membrane sheet in disc form was composed of hydrophilic, low fouling polyvinylidene fluoride (PVDF) polymer with the manifold that collected the permeate flow being located in the single shaft. The measurements of TMP, dissolved oxygen levels, temperature, pH, permeate flux, air scouring flow rates, viscosity and MLSS concentration were all covered in detail in Paul and Jones [17]. Figure 2 shows part of the set-up of this research rig in operation.

**Figure 3:**
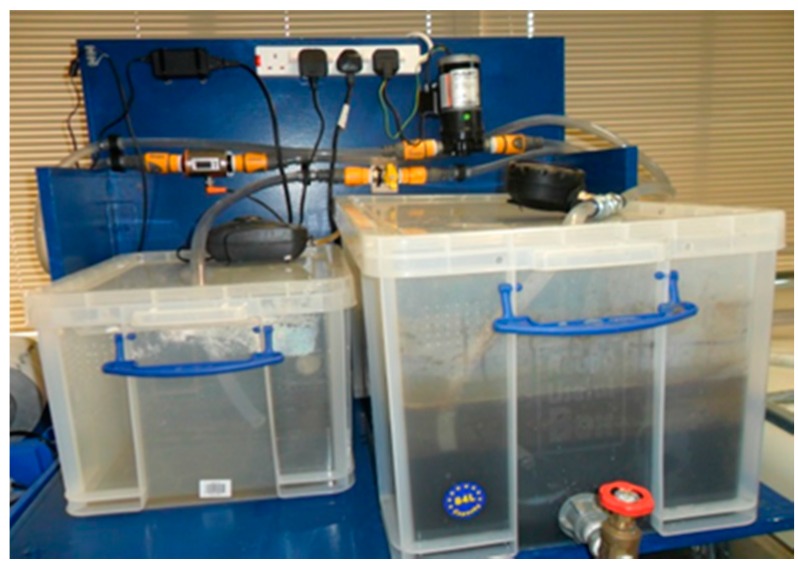
Static square-shaped membrane bioreactor (MBR) system in operation. The square membrane module is located in the larger tank on the right for filtration purposes.

Comparable to above, Figure 3 shows the set-up and operation of a second research MBR rig that was fabricated at Brunel University using a bespoke static square-shaped membrane module (Flexidisks by Avanti Membrane Technology, Walnut, CA, USA). Again in a similar manner to above, this rig generated results and data that in turn were used to validate and test the static MBR fouling model described in Section 2.1. This UF membrane module comprised 20 static membrane flat sheets. Each membrane sheet in square form was also made of hydrophilic, low fouling PVDF polymer. The viscosity of the fluid was measured daily by the aid of rotating viscometers (Rotary-Viscometer ASTM by PCE Instruments UK Ltd., Southampton, UK; and High Shear CAP-2000+ by Brookfield Viscometers Ltd., Essex, UK), while the MLSS concentration was logged continuously by a MLSS analyser (GE-138 MLSS Suspended Solids Sludge Concentration Meter Analyser Monitor by A. Yite Technology Group, Wanchai, Hong Kong).

Table 1 shows this second unit’s membrane dimensioning and a general overview of the operating conditions of the MBR system as provided by the manufacturer.

### 3.2. Plant Operational Regime and Filtration Experimentation

Both MBR plants were initially seeded with activated sludge supplied by Thames Water, UK, and were semi-batch fed a synthetic wastewater made up using a standard recipe to mimic an influent wastewater source. MLSS concentrations were kept between the range of 6.32 g/L and 7.24 g/L by periodic excess sludge wasting. The influents had an average pH between the range of 7.8 and 8.6, and experiments were carried out at a constant room temperature of 23 °C.

**Table 1:** Dimensions and operating conditions of the square-shaped Flexidisks MBR module.

Description	Unit	Values
Individual membrane width	m	0.24
Individual membrane length	m	0.24
Total membrane area	m^2^	1.152
Operating temperature	°C	~5–60 °C
Permeate flux	L·m^2^·h^−1^	>30
TSS (Total suspended solids)	g·L^−1^	>8
TMP (trans-membrane pressure)	bar	(>3)

#### 3.2.1. Shear and Viscosity Experiments for the Rotating MBR

Using the Brookfield rotating viscometer [17] that also acted as a rheometer, measurements were taken following protocols in-line with Yang *et al.* [20] and Ratkovich *et al.* [21]. The proprietary software program for this apparatus was used for system control and collection of data. The sludge was tested at a constant room temperature of 23 °C at a MLSS concentration of 6.32 g/L (although data points at MLSS ranges of 5.2 g/L to 7.3 g/L were also analysed). In addition, the shear stress and viscosity were carefully tested in the shear rate range of 10 s^−1^ to 350 s^−1^. Although full rheology tests were not carried on the activated sludge to ascertain its properties more precisely, the data collected appeared consistent.

#### 3.2.2. TMP Stepping Experiments

A standard TMP stepping procedure was used on the rotating MBR plant as described in detail in Paul and Jones [17]. This same method was also applied to this second rig that employed the square-shaped membrane configuration in its MBR plant. In order to facilitate comparison of operational issues, four TMP steps were carried out for each variation in MLSS concentration for the static square-shaped MBR unit, while for the rotating MBR system only two TMP steps were considered. For the rotating MBR system, TMP steps were carried out at constant TMPs of 15 kPa and 45 kPa. The corresponding initial flow rates for the rotating MBR system were respectively 1.15 × 10^−5^ m^3^/s and 2.45 × 10^−5^ m^3^/s. For the static square-shaped MBR system, the TMP steps up were carried out at constant TMPs of 15 kPa, 30 kPa, 45 kPa and 58 kPa. The corresponding initial flow rates were respectively 1.2 × 10^−5^ m^3^/s, 2.03 × 10^−5^ m^3^/s, 2.5 × 10^−5^ m^3^/s and 3.02 × 10^−5^ m^3^/s. Although data was constantly being logged, for the sake of simplicity and to keep model computation time down to a minimum, only the average data points for every 5 min of filtration time were actually used in the simulation study with the total filtration period being two hours. This meant a total of 25 data points were generated for each individual MLSS concentration.

On unit start-up, for the rotating MBR system, the clean membrane resistance was determined to be 6.26 × 10^11^ m^−1^, while the cake water content, τ, was found to be on average 0.456. Conversely on start-up, the square-shaped MBR system had a pristine membrane resistance of 4.55 × 10^11^ m^−1^.

## 4. Results and Discussions

### 4.1. Rotating MBR Model Validation with Hydrodynamic and Shear Effects

#### 4.1.1. Shear and Viscosity Relationship

A major parameter included in this model formulation was combined shear due to both rotation and aeration as this is a unique feature of this type of MBR configuration. To that end, the viscosity of the MLSS at 6.32 g/L concentration (points at 7.24 g/L were also considered) was measured at different shear rates. The rotational speed of the spindle was 20 RPM and this equated to a shear rate of 26 s^−1^. Although full rheology tests were not carried on the activated sludge to ascertain its properties more precisely, the data collected appeared consistent. Results indicated that the fluid’s viscosity had decreased much faster and at a higher shear rate (by over 32%). That was expected since the calculated radial Reynolds number (R_eNN_) showed that the flow was laminar during MBR operations (*i.e.*, R_eNN_ of 8.26 × 10^3^ which is less than the 2 × 10^5^ limit). It should be noted that rotating MBR systems can handle shear rates up to 2 × 10^5^ s^−1^. However, the range of shear rate tests were kept minimal since the pilot MBR in question only operated at very low RPMs. Furthermore, it should be noted that since activated sludge is a shear thinning fluid [7], the rheological measurement range must be kept in the laminar region otherwise the rheometer’s output becomes increasingly hard to interpret.

The viscometer’s output was used to determine parameters *m* and *n* for this activated sludge. These parameters are the coefficients governing the shear rate and viscosity respectively and are related by a logarithmic law. Since the shear equation [17] obeys a power law, it naturally follows that the logarithmic of said equation would produce a polynomial of order one (*i.e.*, a straight line). Consequently, the parameters *m* and *n* were specifically determined by plotting the logarithm of viscosity against the logarithm of the shear rate using a linear curve fitting process in the Matlab and Excel software packages.

**Figure 4:**
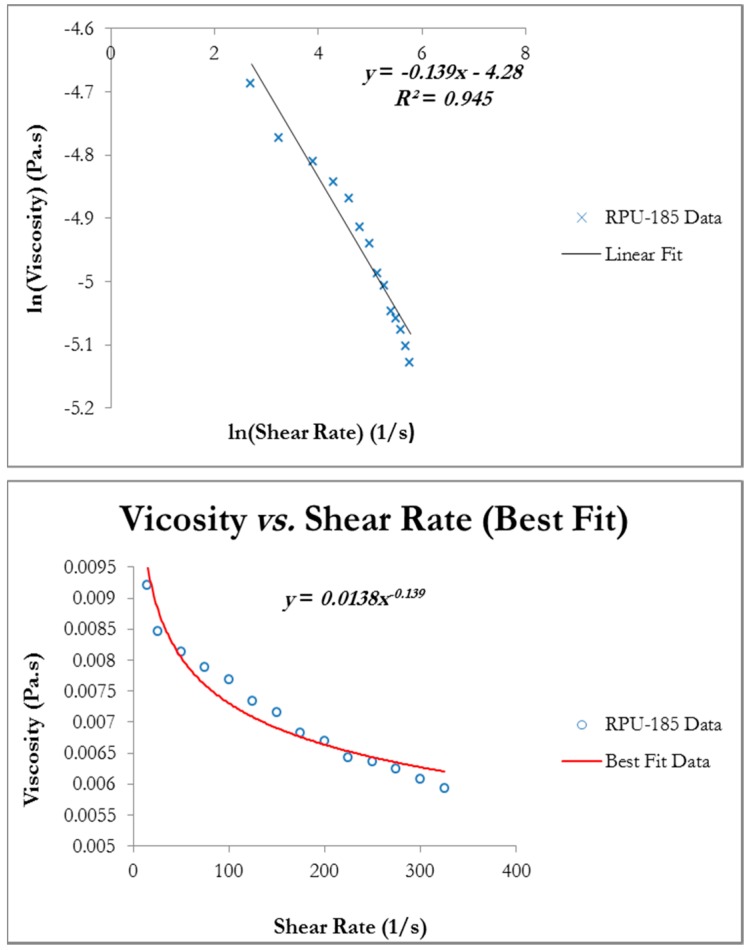
Viscosity plotted against shear rate for rotating MBR system RPU-185.

Figure 4 shows the viscosity plotted against the shear rate with the solid line being the best fit. The coefficient *m* was found to be 0.0138 whilst *n* gave a value of 0.861. The coefficient of determination for the linear fit process was 0.945, indicating a respectable model fit since the sum of the squared residuals was also minimised. A value of *n* less than one was a clear indication that the fluid had deviated from Newtonian behaviour. Nevertheless, it is worth noting that higher MLSS concentrations are associated with high viscosity that, in turn, strongly hinders mass transfer leading to increases in membrane fouling [20].

#### 4.1.2. Rotating MBR Model Validation with Hydrodynamic Regime

In this developed rotating fouling model parameters α, β (σ*_a_* is proportional to β and is therefore less significant) and a combination of *f’.R’*, *R_bo_*, *g_o_* and *k_Ab_*; each solely and respectively contributed to pore blocking, pore constriction and cake filtration [15].

As described in the earlier study by Paul and Jones [17], the air scouring coefficient, α*_v_*, and the resistance distribution, δ, were obtained via sensitivity analysis. The values found were respectively 0.0292 and 4.6 × 10^−4^ m^−1^ for α*_v_* and δ. The air scouring flow rate of 3.55 × 10^−4^ m^3^/s was also kept the same for all simulations. Due to varying fluxes, the values of the term *k_ω_* were obtained via sensitivity analysis and are shown in Table 2. These above determined values were kept constant and used in all subsequent simulations to calculate the best fit values for this fouling model. To ensure validity, only the six most important parameters pertaining to all three fouling mechanisms were used for curve fitting during simulations and these *were f’.R’,* α, β, *R_bo_/R_m_,* *g_o_* and *k_Ab_*. These best fit simulation values were attained by minimising the sum of squared residuals between the model and the collected experimental data. This was used in conjunction with the “Genetic Algorithm” function in the Matlab software package with an initial population large enough for the data set used to converge to the minimal possible error. The simulations were performed for each TMP at respectively 15 kPa and 45 kPa for MLSS concentrations of 6.32 g/L and 7.24 g/L. The term σ*_a_* was calculated upon obtaining the optimal fitting value of β since the membrane pore size was known. 

**Table 2:** Simulations best fit model parameters for rotating MBR system RPU-185.

Parameters	Unit	15 kPa TMP Step	45 kPa TMP Step
*g_o_*		79.59	24.76
*k_Ab_*		0.025	26.57
*R_bo_/R_m_*		0.206	0.324
*f’R’*	m/kg	0.248 × 10^9^	199.73 × 10^9^
α	m^2^/kg	0.118	1.157
β	kg	0.893	0.589
σ*_a_*	kg·m^3^	6.816 × 10^−^^17^	4.497 × 10^−^^17^
*m*	Pa·s ^n^	0.0138 ± 0.02	0.0138 ± 0.02
*n*		0.861 ± 0.01	0.861 ± 0.01
*k*_ω_	rad^−1^	2.812 × 10^−5^	5.991 × 10^−5^

In summary, the values of shear parameters *m* and *n* are within acceptable range as they are in-line with those found in earlier work [17]; however, these values change at a steady rate depending on the range of MLSS concentrations used and since high viscosities are associated with high MLSS, the likelihood of cake filtration (or cake formation) occurring is drastically increased at high TMPs. The values of α*_v_*, δ and *k_ω_* are also adequate since the same conditions found by Paul and Jones [17] were used during experimentation. Moreover, fitting parameters *f’.R’*, α, β, *R_bo_/R_m_*, *g_o_* and *k_Ab_* all vary based on the TMP used but stay constant within simulated MLSS concentration range of 6.32 g/L and 7.24 g/L. These best fit values obtained from simulation appear fairly reasonable since they are similar in scope with former work completed by Paul and Jones [17]. Finally, these best fit values also represent an accurate portrayal of experimental data collected and the dominant fouling mechanisms. 

**Figure 5:**
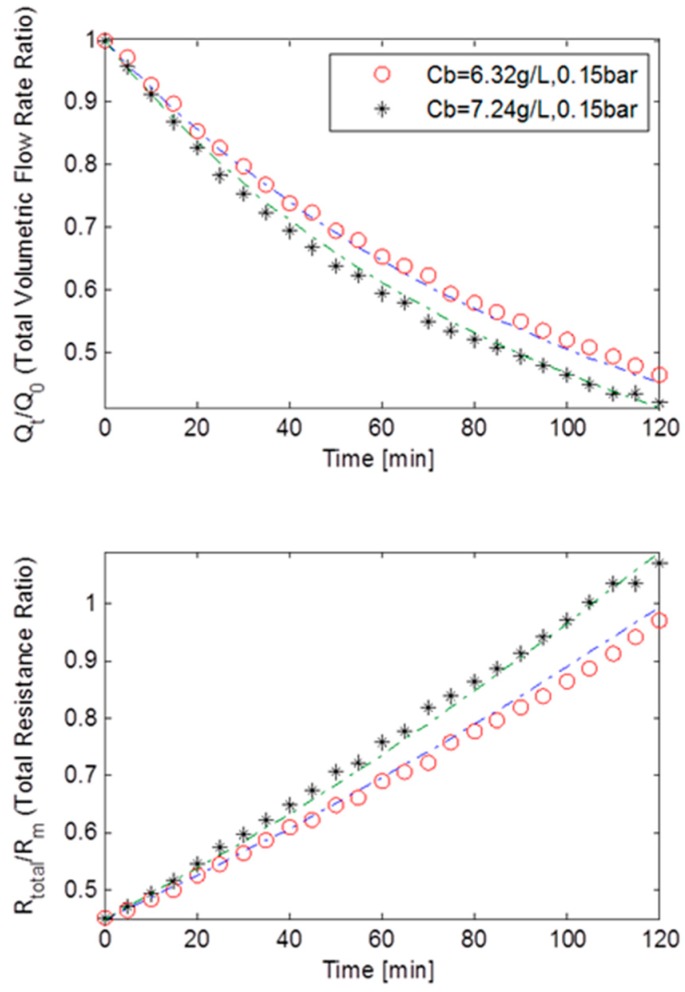
Flux decline and total resistance for TMP step of 15 kPa for the rotating MBR system RPU-185 with best model fits.

Figure 5 displays the TMP step data as normalised volumetric flow rates and the total resistance ratios plotted against the filtration time at a constant TMP of 15 kPa for MLSS concentrations of 6.32 g/L and 7.24 g/L for the rotating membrane system RPU-185; with the dashed lines representing the best fit simulation solution. It should be noted that the total resistance was calculated using Darcy’s Law. A sharp decrease in flux of 56% can be observed at roughly both MLSS concentrations. This is in-line with the results produced by the earlier study by Paul and Jones [17] that found the rate of decrease of flux is consistent and steady unless the TMP is dramatically increased. The trend shown by the both sets of curves is sufficient to suggest that fouling occurring during filtration was caused by a combination of all three classical fouling mechanisms. This is further supported by the fact that the resistances’ curves displayed a linear best fit. However, as shown in Table 2, it is found upon closer inspection that a relatively small *R_bo_* factor and a big cake removal factor, *g_o_*, indicated a weak cake layer formation which is reinforced by a small *f’.R’* (fractional amount of total foulants multiplied by specific cake layer resistance) and a very small *k_Ab_* (blocked pores area constant). Thus, the combination of parameters *f’.R’*, *R_bo_*, *g_o_* and *k_Ab_*, implied that cake filtration was less prevalent which was anticipated since at low TMP of 15 kPa, cake formation is expected to be less dominant [17]. Furthermore, the pore constriction parameter, β, being roughly eight times bigger than the pore blocking parameter, α, ultimately suggested that fouling was dominated by pore constriction (as β >> α).

At MLSS concentrations of 6.32 g/L and 7.24 g/L and constant TMP of 45 kPa, the starting initial flow rate was 2.45 × 10^−5^ m^3^·s^−1^. Experimental data showed that at MLSS concentration 6.32 g/L, the flux had diminished drastically and dropped by more than 75%. A similar reduction was detected at MLSS concentration of 7.24 g/L. This meant that not only had the initial flux increased at higher TMPs, but also that the flux decline rate had increased at greater pressure. These findings are in-line with theory since a membrane is likely to foul more quickly when approaching or exceeding critical flux [9,17].

**Figure 6:**
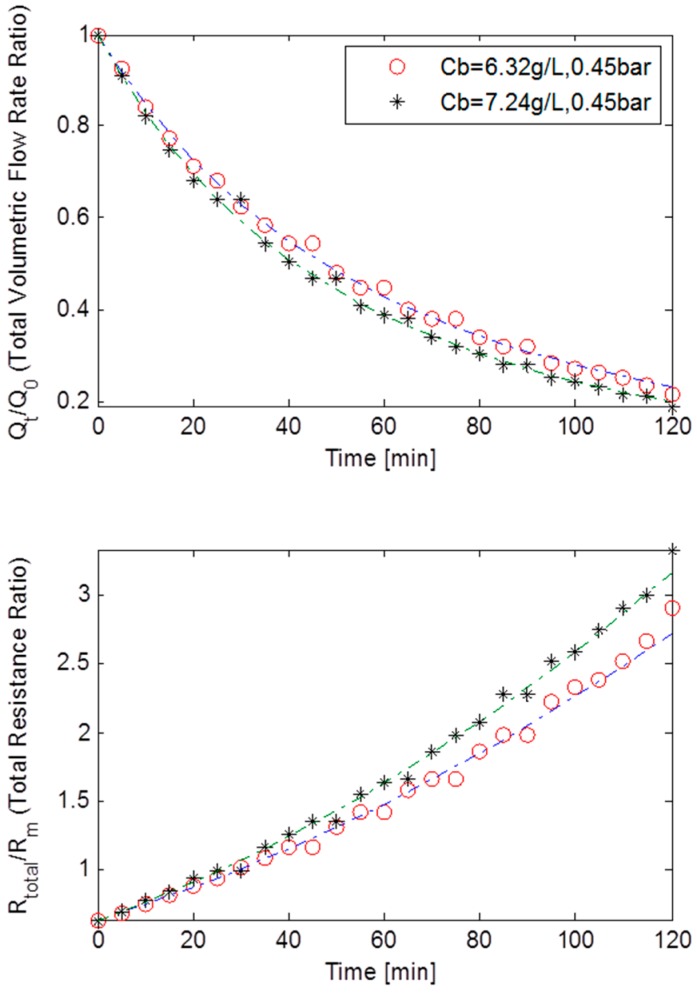
Flux decline and total resistance for TMP step of 45 kPa for the rotating MBR system RPU-185 with best model fits.

Figure 6 shows the normalised volumetric flow rates and the total resistance ratios plotted against the filtration time at constant TMP of 45 kPa, for MLSS concentrations of 6.32 g/L and 7.24 g/L for the rotating membrane system RPU-185. The massive drop in flux also resulted in the total resistance increasing exponentially. This strongly indicates that the fouling in this case was dominated by both pore blocking and cake filtration although the combined effect of all three fouling mechanisms on the total fouling cannot be precisely extrapolated. Again as presented in Table 2, a relatively bigger *R_bo_* and *f’.R’* when compared to the data from the constant TMP of 15 kPa step, coupled with near equally sized *g_o_* and *k_Ab_*, indicated that an adequately strong cake layer was formed. However, with α being roughly twice as big as β, the pore blocking fouling mechanism must have also had a significant impact on fouling. Thus, it is thought that the bulk of the fouling was dominated by both pore blocking and cake filtration.

### 4.2. Static MBR Model Validation with Hydrodynamic and Non-Shear Effects

The flow regimes during filtration processes were all laminar which were well within expectations since calculated R_eNN_ values were much less than 2 × 10^5^.

As mentioned in Section 4.1.2, in this static fouling model, parameter α solely contributed to pore blocking; parameter β wholly contributed to pore constriction and finally a combination of parameters *f’.R’*, *R_bo_*, *g_o_* and *k_Ab_*, all served to define the cake filtration.

The aeration rates for all the data sets for the static MBR system were similar in scale to that of the rotating MBR system operated under lab scale conditions. Hence, similar constant values for air scouring coefficient, α*_v_*, (*i.e.*, 0.0292), and resistance distribution factor, δ, (*i.e.*, 4.6 × 10^−4^ m^−1^) were used during all simulations. Additionally, since the static square-shaped MBR system was not operated under rotation, the shear effects parameters *m*, *n* and *k_ω_* were removed and not used during all simulations. As discussed in section 4.1.2 and also as applied to the static fouling model, the key parameters necessary for analysis, curve fitting, and fouling mechanisms determination were *f’.R’*, α, β, *R_bo_/R_m_*, *g_o_* and *k_Ab_*. These output values were determined in Matlab using the “Genetic Algorithm” function in concurrence with the minimised sum of squared residuals between the fouling model and experimental data. Simulations were swiftly performed for each TMP at correspondingly 15 kPa, 30 kPa, 45 kPa and 58 kPa for MLSS concentrations of 6.32 g/L and 7.24 g/L. 

Best fit parameters *f’.R’*, α, β, *R_bo_/R_m_*, *g_o_* and *k_Ab_* as presented in Table 3, all appear to be in sensible agreement with prior conducted work [15,17]. A justification for this is that despite variations depending on the TMP used, these values remained constant within simulated MLSS concentrations range of 6.32 g/L and 7.24 g/L. 

In Table 3, when comparing pore blocking parameter, α, at different data sets, its lowest value of 0.094 is found for the Avanti static square-shaped MBR system for TMP step of 15 kPa. This suggests that the pore blocking’s effect on fouling was minimal during filtration. Moreover, this is justified since its corresponding pore constriction parameter, β, is much bigger. Thus, a possible conclusion is that at lower TMPs, pore blocking is less likely to dominate fouling, which is also in-line with Paul and Jones [17] findings. In contrast, the highest value of the pore blocking parameter, α, is 0.907 and is found for the Avanti static square-shaped MBR system for TMP step of 45 kPa. It implies that pore blocking was one of the dominant fouling mechanisms during filtration, which is reasonable since its corresponding pore constriction parameter, β, is much smaller. Therefore an inference is that at very high TMPs, pore blocking is more likely to be one of the dominant fouling mechanisms, which again happens to be in-line with prior work [17].

**Table 3:** Simulations best fit model parameters including hydrodynamic effects.

Optimised Parameters	*f’R’* (m/kg)	α (m^2^/kg)	β (kg)	σ*_a_* (kg·m^3^)	*R_bo_/R_m_* (−)	*g_o_* (−)	*k_Ab_* (−)
Duclos-Orsello *et al.* [15] data	1.81 × 10^11^	0.576	2.538	19.4 × 10^−17^	0.168	24.58	1.46
Pilot MBR unit at Coors (UK) data	1.76 × 10^11^	0.122	0.289	2.21 × 10^−17^	0.159	27.24	0.108
Static MBR unit (15 kpa)	0.834 × 10^11^	0.094	0.492	3.79 × 10^−17^	0.126	63.75	9.36
Static MBR unit (30 kpa)	0.947 × 10^11^	0.184	0.305	2.33 × 10^−17^	0.692	10.99	2.032
Static MBR unit (45 kpa)	108.8 × 10^11^	0.907	0.486	3.78 × 10^−17^	0.526	15.36	14.23
Static MBR unit (58 kpa)	32.01 × 10^11^	0.221	0.212	1.62 × 10^−17^	0.457	29.66	1.26

When comparing pore constriction parameter, β, at various data sets, its lowest value of 0.212 is found for the Avanti static square-shaped MBR system for TMP step of 58 kPa (see Table 3). As such, it can be inferred that pore constriction had a lesser impact on fouling or was equally as influential as other fouling mechanisms. Since its equivalent pore blocking parameter, α, is almost equal in value (though still a bit less), it can be argued that both pore blocking and pore constriction had almost equal importance in the occurrence of fouling. Consequently, a conclusion that can be drawn is that at higher TMPs, pore constriction is less likely to be dominant [17]. On the other hand, the highest value of pore constriction, β, is 2.538 and is found for the Duclos-Orsello *et al.* [15] data set for constant TMP of 14 kPa. It therefore implies that pore constriction was one of the dominant fouling mechanisms during filtration. Additionally, this is justified since its corresponding pore blocking parameter, α, is almost five times smaller. Accordingly, it can be concluded that at lower TMPs, pore constriction is more likely to dominate fouling [17]. 

As shown in Table 3, when comparing the combination of parameters *f’.R’* (fractional amount of total foulants multiplied by specific cake layer resistance), *R_bo_* (initial resistance of solids deposit), *g_o_* (cake removal factor) and *k_Ab_* (blocked pores area constant), which all pertains to cake filtration, its lowest combination is found for the Avanti static square-shaped MBR system for TMP step of 15 kPa. This suggests that cake formation (and by extension cake filtration) was fairly weak and less influential during fouling. Thus, at lower TMPs, cake filtration is expected to be less dominant and prevalent during fouling [17]. Conversely, the highest combination of parameters *f’.R’*, *R_bo_*, *g_o_* and *k_Ab_*, is found for the Avanti static square-shaped MBR system for TMP step of 45 kPa. It implies that cake filtration was fairly strong and more significant during fouling. As such, it can be deduced that at high TMPs, cake filtration is expected to be one of the dominant fouling mechanisms [17].

Overall, the calculated model parameter values gave very respectable fits when compared to the original data collected for the static square-shaped MBR system, although extreme MLSS concentration values of 8 g/L and 12 g/L respectively for the Duclos-Orsello *et al.* [15] data, and the Coors (UK) data [16], gave rather poor fits. This is largely as expected since the sludge rhealogical effects which themselves are volatile will dominate all membrane fouling mechanisms in unpredictable ways especially at high MLSS concentrations with associated high viscosities. Additionally for the static square-shaped MBR system it can be seen that at higher TMPs, the simulation fit progressively deteriorated as the flux declined rapidly. Thus, at a TMP step with constant TMP of 45 kPa, the simulation’s fit was extremely poor and, by extension, was poor for the 58 kPa case as well. This situation is less prevalent for the rotating MBR system, as both air scouring and rotational shear contribute to the reductions in fouling. 

In order to validate the static model, two distinct sets of data were used. The first set was collected from the study carried out by Duclos-Orsello *et al.* [15], and the other was data provided by a pilot MBR plant operated at Coors (UK) [16]. It should be noted that, for the Coors (UK) data, the viscosity (Pa·s) was calculated from the MLSS data provided and by using Equation (19) [20].

(19) $$\mu =0.0126.{\left(C_{\mathrm{MLSS}}\right)}^{1.664}.e^{\frac{E}{R_{g}.(T_{\mathrm{room}}+273.15)}}$$

Additionally, four sets of data all obtained via a TMP stepping procedure using the static square-shaped MBR system were used to validate this static fouling model. The data was collected at TMP steps with constant TMPs of 15 kPa, 30 kPa, 45 kPa and 58 kPa respectively. There were obviously no induced shear effects to take into account, which simplified the entire simulation procedure since only the hydrodynamics effects were included with appropriate parameters and coefficients used during simulation runs.

**Figure 7:**
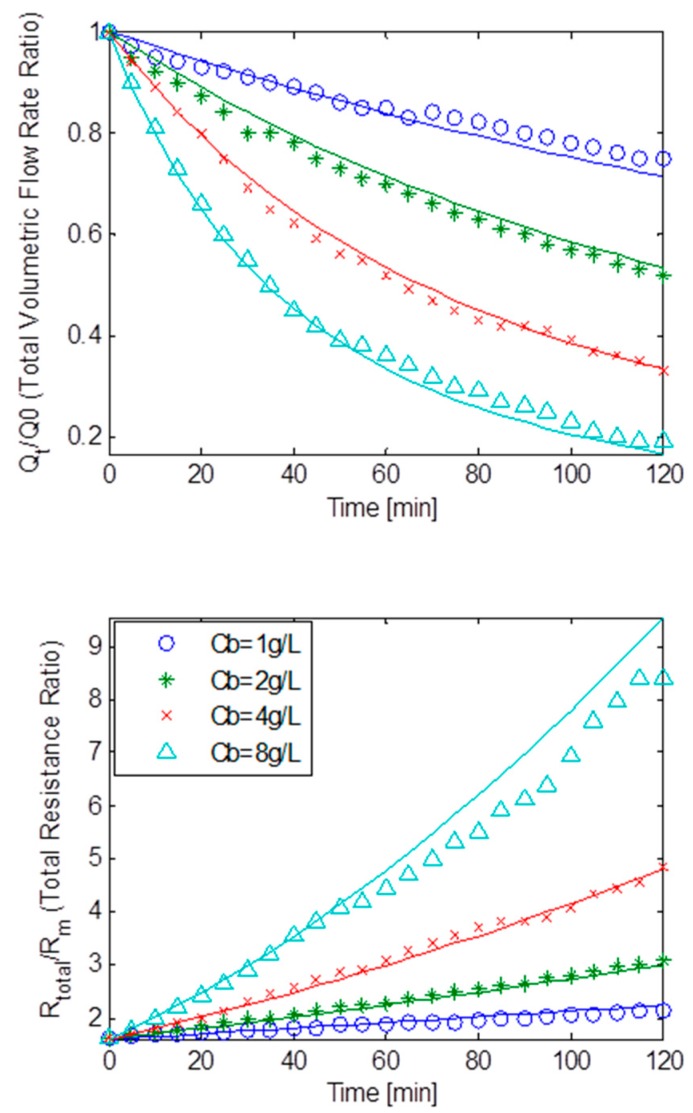
Flux decline and total resistance data obtained from Duclos-Orsello *et al*. [15] with best model fits.

Figure 7 depicts the TMP step data as normalised volumetric flow rates and the total membrane resistance ratios plotted against the filtration time at a constant TMP of 14 kPa for MLSS concentrations varying from 1 g/L to 8 g/L based upon the Duclos-Orsello data; with the solid lines representing the best fit simulation solutions. Again, the total resistance was acquired via Darcy’s law. It was immediately noticeable that at the highest MLSS concentration of 8 g/L, the flux decline was much greater than for all lesser concentrations. In fact it was an actual decrease of about 84% making the total membrane resistance climb steeply for this entire filtration event. This indicated that fouling was dominated by pore constriction even though all three types of fouling mechanisms were in operation to a greater or lesser degree. This conclusion appears justifiable since the calculated pore constriction parameter, β, was found to be roughly four times the size of the pore blockage parameter, α (when referring to Table 3). Additionally, a weak deposit layer as depicted by *R_bo_*, and a big cake removal factor, *g_o_*, indicated a fairly weak cake layer formation which is reinforced by a smaller *f’.R’* factor and a small blocked pores area constant, *k_Ab_*. Thus, the combination of parameters *f’.R’*, *R_bo_*, *g_o_* and *k_Ab_*, implied that cake filtration was relatively less significant during fouling which was expected for such low TMP operations. These finding seem to concur with the results found in the original study by Duclos-Orsello *et al.* [15]. As with the original study, at bulk concentrations of 1 g/L and 2 g/L the resistances and the volumetric flow rates seemed essentially linear.

**Figure 8:**
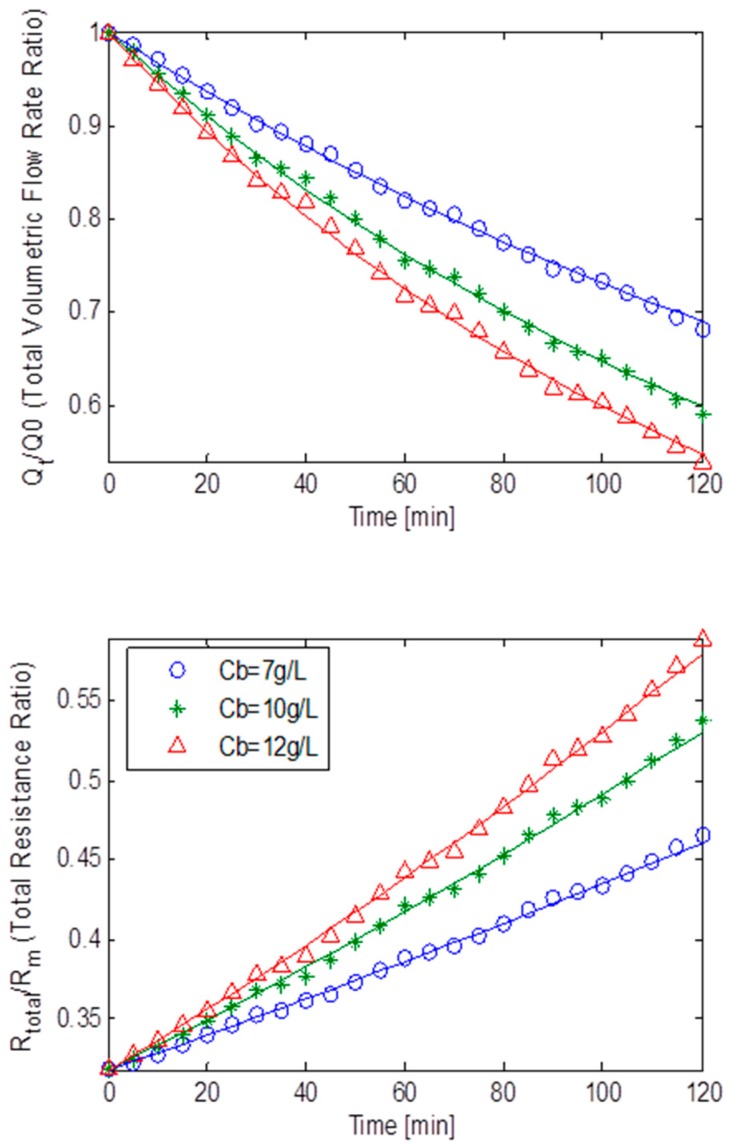
Flux decline and total resistance data obtained from pilot MBR plant located at Coors (UK) with best model fits.

Figure 8 shows the normalised volumetric flow rates and the total resistances ratios versus the filtration time at a constant TMP of 18 kPa with initial flow rate of 1.24 × 10^−5^ m^3^/s, for normalised MLSS bulk concentrations of 7 g/L, 10 g/L and 12 g/L respectively for the data supplied by the pilot MBR unit operated at Coors (UK) [16]. This MBR system’s data set had typically high MLSS values that are usually associated with high overall fluid viscosities. This suggested that the flux was expected to decline rapidly at the highest MLSS concentrations. Indeed it is found that at a MLSS concentration of 12 g/L, the flux had declined almost linearly by 63%. High MLSS concentrations at respectively 7 g/L and 10 g/L exhibited this similar declining behaviour albeit at slightly reduced rates of 59% and 61% respectively. Although slightly below theoretical expectations due to arguably a low TMP regime, the linear increase in resistances was probably caused by the combined effect of all three fouling mechanisms occurring simultaneously with pore constriction being slightly more dominant. As shown in Table 3, and as expected for a low TMP operational regime, an analysis of the combination of parameters *f’.R’*, *R_bo_*, *g_o_* and *k_Ab_*, indicated that cake formation was moderately weak. Moreover, a much bigger β than α suggested that pore constriction was somewhat more prevalent in fouling.

**Figure 9:**
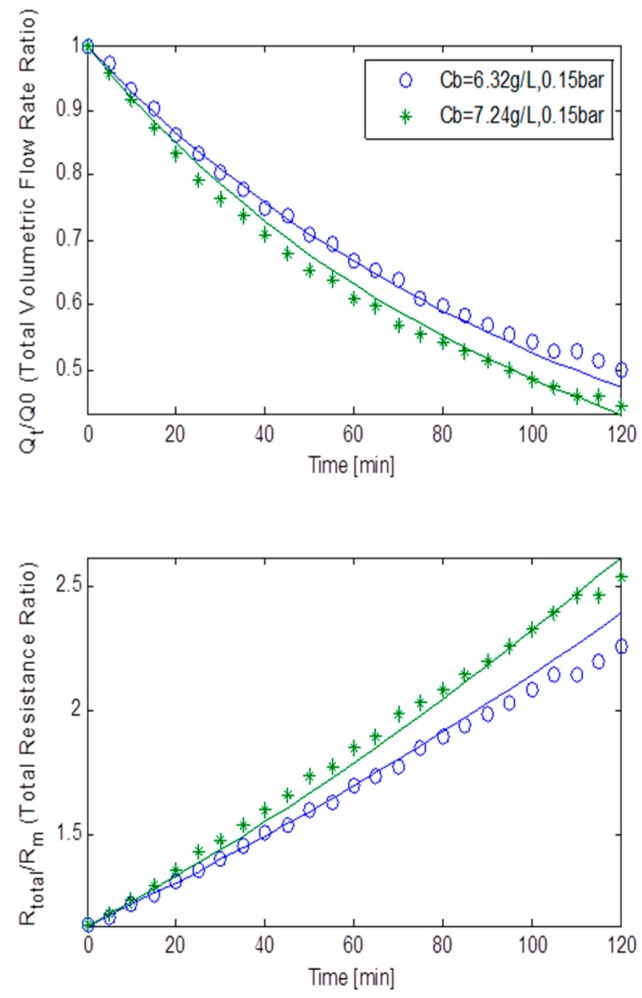
Flux decline and total resistance for TMP step of 15 kPa for the static square-shaped MBR system with best model fits.

Figure 9 displays the normalised volumetric flow rates and the total resistances ratios plotted against the filtration time at a constant TMP of 15 kPa, for MLSS concentrations of 6.32 g/L and 7.24 g/L for the static square-shaped MBR system. These MLSS concentrations of 6.32 g/L and 7.24 g/L had their fluxes decline at a steady rate and go down approximately by 64%. The resistances displayed an almost linear trend, suggesting fouling was caused by the combination of all three fouling mechanisms. As observed in Table 3, a fairly small *R_bo_* factor and a big *g_o_*, indicated the formation of a weak cake layer which was further reinforced by a small *f’.R’* and *k_Ab_*. As a result, the combined parameters *f’.R’*, *R_bo_*, *g_o_* and *k_Ab_*, suggested that cake filtration was less dominant during fouling which was anticipated since at a low TMP of 15 kPa, cake formation is expected to be less prevalent [17]. Furthermore, β being roughly five times bigger than α, ultimately suggested that fouling was mostly caused by pore constriction. 

As can be seen, after two hours of ultra-filtration at MLSS concentrations of 6.32 g/L and 7.24 g/L, and at a constant TMP of 30 kPa, a similar flux decline comparable in size to the 15 kPa TMP case was observed. The rate of decline was steady and this gradual drop was expected since it was in-line with critical flux theory [9].

**Figure 10:**
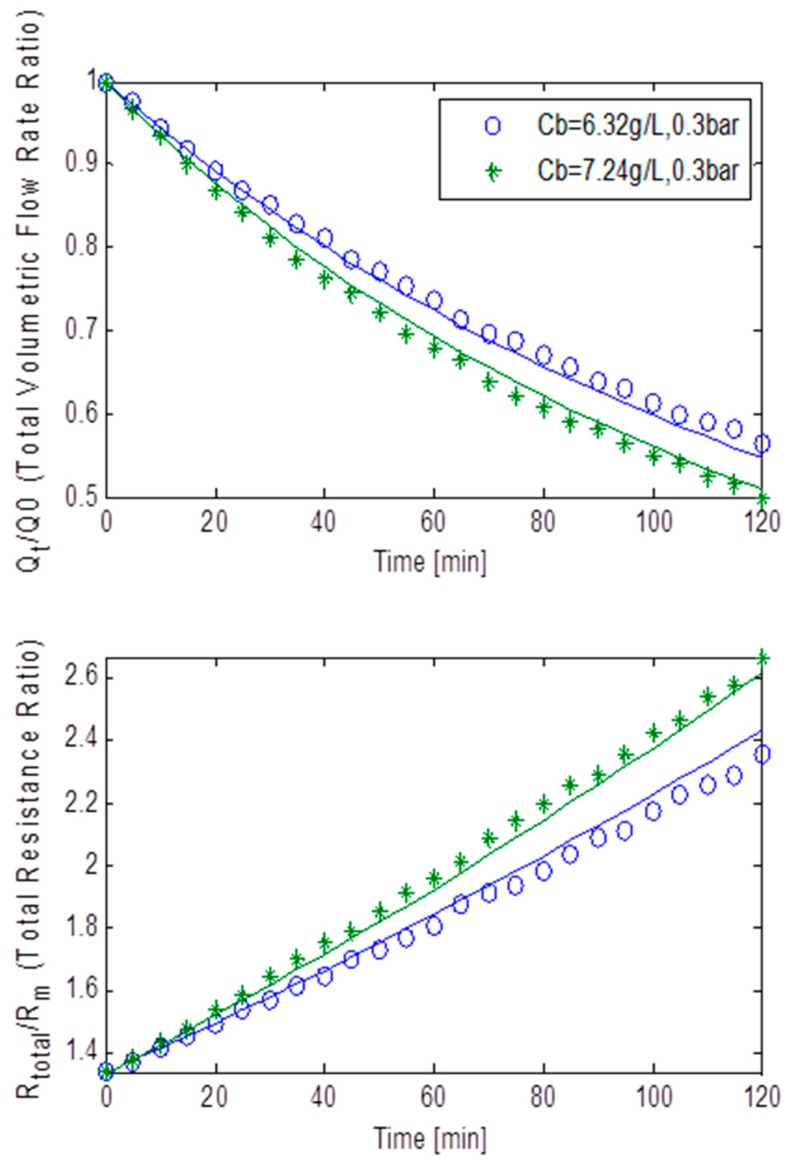
Flux decline and total resistance for TMP step of 30 kPa for the static square-shaped MBR system with best model fits.

Figure 10 portrays the normalised volumetric flow rates and the total resistances ratios plotted against the filtration time at a constant TMP of 30 kPa, for MLSS concentrations of 6.32 g/L and 7.24 g/L for the static square-shaped MBR system. The resistance-time plot, once again, seems to indicate that fouling could be attributed to the combined effect of all three mechanisms. Again as shown in Table 3, a fairly big *R_bo_* and a small *g_o_*, indicated a reasonably adequate cake layer formation which was supported by a bigger *f’.R’* factor and a small *k_Ab_* when compared to the data from the constant TMP step of 15 kPa,. Consequently, the combination of parameters *f’.R’*, *R_bo_*, *g_o_* and *k_Ab_*, suggested that cake filtration was adequately prevalent during fouling. In addition α being much smaller than β, all seemingly implied that most of the fouling was dominated by both pore constriction and cake filtration.

**Figure 11:**
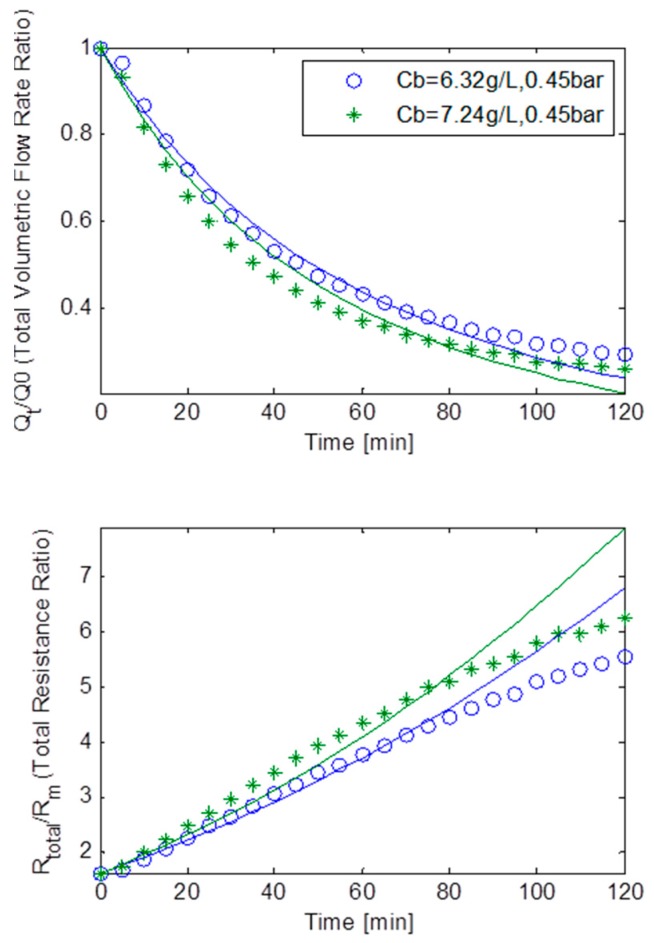
Flux decline and total resistance for TMP step of 45 kPa for the static square-shaped MBR system with best model fits.

Figure 11 represents the effects of the fouling behaviour of the static square-shaped MBR system, using both the normalised volumetric flow rates and total resistance ratios for MLSS concentrations of 6.32 g/L and 7.24 g/L at a constant TMP of 45 kPa. Due to a colossal drop in flux at this high TMP, the total resistance increases at an exponential rate. It is noticeable that the best fit curve at this high TMP is extremely poor (especially after 80 min), but however the curve’s trend is of the right scale and in the right direction to allow analysis of the fouling behaviour that is occurring. At first glance, analysis would suggest that fouling may be due to all three fouling mechanisms, but it can be argued that fouling was mainly dominated by cake filtration and pore blocking. A reasonably big *R_bo_* and *f’.R’* coupled with similar sized *g_o_* and *k_Ab_*, all strongly indicated that a fairly large cake layer was formed (as seen Table 3). However, with α being roughly twice as big as β, it can be concluded that the pore blocking fouling mechanism was also prevalent during fouling. Hence, the bulk of the fouling was dominated by both cake filtration and pore blocking.

**Figure 12:**
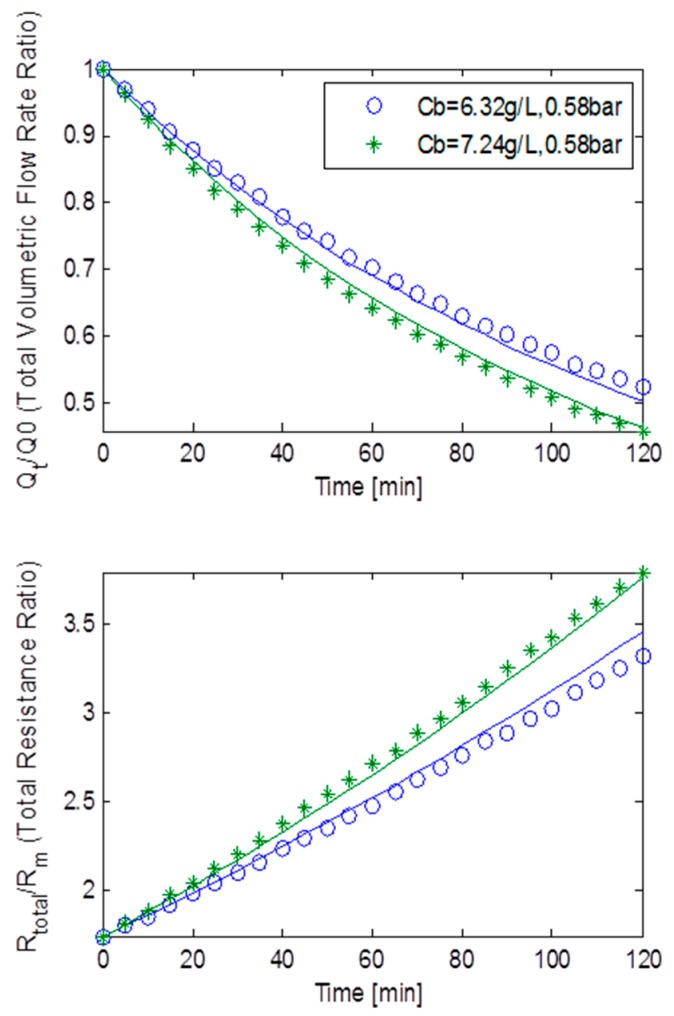
Flux decline and total resistance for TMP step of 58 kPa for the static square-shaped MBR system with best model fits.

Figure 12 shows the fouling behaviour of the static square-shaped MBR system, using both the normalised volumetric flow rates and total resistance ratios for MLSS concentrations of 6.32 g/L and 7.24 g/L at a constant TMP of 58 kPa. The total resistance seemingly increases in a linear fashion with filtration time albeit at a high rate. This is probably because fouling was caused by all three mechanisms happening simultaneously. Parameters α and β being of almost equal size suggests that neither of the two fouling mechanisms was dominant (Table 3). Furthermore, a moderately big *R_bo_* and *f’.R’* when compared to the data from the constant TMP step of 15 kPa, together with an adequate *g_o_* value and a small *k_Ab_* value, all indicated that a fairly decent size cake layer was formed. Accordingly, all three fouling mechanisms were of equally great importance during fouling. 

### 4.3. Comparison of Simulation Results Generated from Rotating and Static MBR Models

After every simulation done in Matlab, a mean of minimised residuals (*i.e*., mean fitness) and the best minimised residuals (*i.e*., best fitness), are automatically calculated. These can in turn be used to accurately determine how well a simulation fit was conducted. Needless to say, the smaller these values, the better analysis can be conducted. Using these facts, a comparison between both models (static and rotating) and their fitness values will be conducted and some conclusions drawn.

When comparing and fitting the data obtained from the rotating MBR system to that obtained from the static square-shaped MBR system, the most prominent difference found for the latter case is the poor fit between the experimental data and the simulation runs. Table 4 summarises the best fitness values after simulations for both systems for the same two TMP steps at constant TMPs of 15 kPa and 45 kPa respectively. As can be seen, the best fitness values for the rotating MBR system are all much smaller than those for the static square-shaped MBR system. In fact the best fitness values for the latter are roughly ten times greater than that for the rotating MBR system. The difference here can be probably attributed to the fact that the shear effects are instrumental in reducing the overall fouling for the rotating MBR system while the static square-shaped MBR system is only aided by standard air scour alone. This concurs with critical flux theory and the area-loss model that explains any rapid TMP jump phenomenon [9], since greater fouling will lead to greater uneven cake build up, leading to loss of clean membrane surface, meaning that locally critical flux could be exceeded, thus promoting the likelihood of rapid declines in fluxes at higher TMPs at unpredictable rates.

**Table 4:** Statistics with fitness values from simulations as computed in Matlab.

Fitness Statistics	Avanti Rotating MBR		Avanti Static Square-Shape MBR	
Fit Parameters	15 kPa TMP	45 kPa TMP	15 kPa TMP	45 kPa TMP
Best fitness (−)	0.0361	0.0294	0.346	1.293
Mean fitness (−)	0.0849	0.0672	0.968	4.541

In simple terms, this means that for the same constant TMP regime for both systems, the fouling build up expected on the rotational MBR system would be less with a reduced likelihood of local critical flux being exceeded. Thus, data sets obtained under this system would be expected to have flux declines occurring in more predictable and consistent ways, and at reduced levels even at higher TMPs when compared to the static MBR system. Consequently, it would be expected that more consistent and less variable data would give rise to better simulation fits for the rotating MBR system, and conversely produce a much less reasonable and agreeable fit for the static MBR system even though both were manufactured by the same company, using the same pipe manifolds, same membrane materials, same membrane pore sizes, and same spacing between individual membrane sheets. 

**Figure 13:**
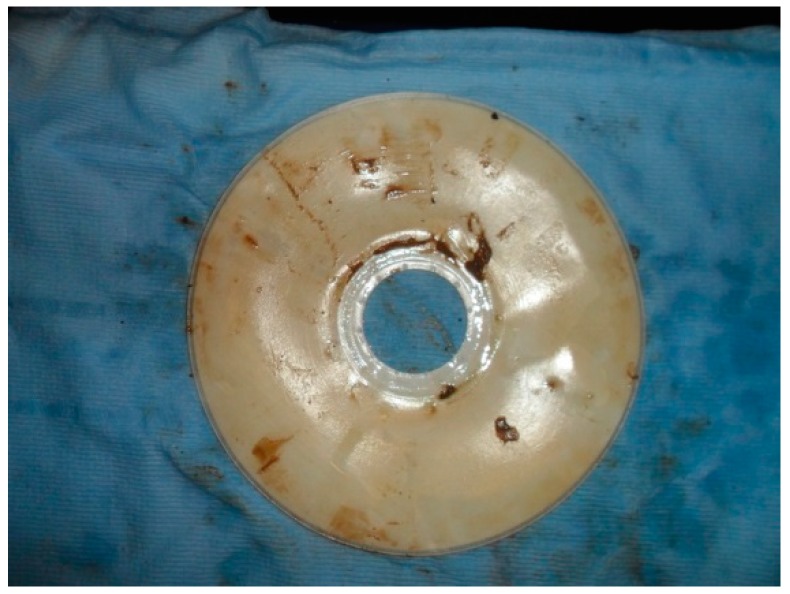
Impact of fouling on the rotating MBR system—even caking is observed.

**Figure 14:**
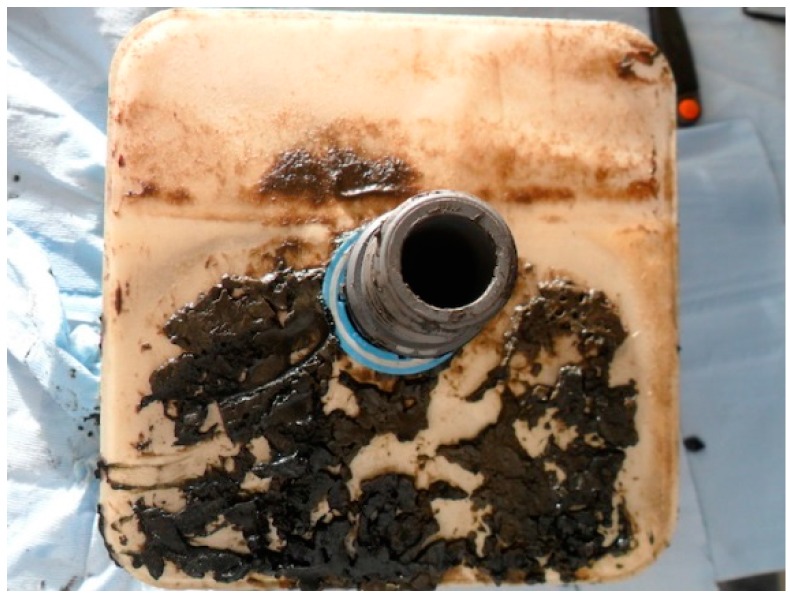
Impact of fouling on the square-shaped MBR system—uneven caking is observed.

A logical conclusion from these all these results is that for the rotating MBR system, its additional rotation shear evens out the cake formation on the membrane surface while for the square-shaped MBR system, the cake distribution is highly uneven and less predictable. In physical terms this hypothesis can be confirmed, since it is very evident when carrying out membrane autopsies for both system types, it is clear that huge cake build up occurs on the membrane surface for the static MBR system. Figure 13 and Figure 14 show the actual typical caking patterns observed on both types of membrane sheets when they were individually removed from the bioreactor. It must be particularly noted in Figure 14, the heavy fouling that is occurring on the bottom half of the square membrane sheet where the air scour effect is least prevalent due to the small distance travelled by the coarse air bubbles used. All of these simulation results and actual physical observations, indicate that the rotating MBR system may be a more preferable option if reduced fouling was a key need in the system design.

## 5. Conclusions

Both MBR model types were extensively tested using short term data generated by both pilot units and medium term data from a similar pilot unit operated at Coors (UK) [16]. Reasonable agreement was reached between expected results and simulation outputs, although the rotating MBR system outperformed the static MBR system when both were operated under similar short term TMP stepping regimes, with the former giving much better model fits than the latter.

The study concluded that a rotating MBR system could increase flux throughput by a significant amount when compared to a similar static system although there are obvious additional capital and operational cost implications. It is thought that although the slowly rotating spindle induces a very weak crossflow shear, it is still able to even out cake build up across the membrane surface, thus reducing the likelihood of localised critical flux being exceeded and lessening the potential of inducing any subsequent and unpredictable TMP jump phenomenon [9].

Even though the rotating pilot unit exhibited reduced fouling when compared to the static square version, it must be remembered that due to the patented edge sealing method used by Avanti Technology, USA, there are still potential scaling up issues to be addressed for both these MBR configurations.

This critical research work into novel MBR processes for wastewater treatment and recycling adds to the body of research work into the overarching theme of environmental systems engineering. Follow-on work in this area will attempt to confirm and extend these initial findings.

## Abbreviations

*A_b_*, blocked membrane area (m^2^);

*A_u_*_0_, initial unblocked area (m^2^);

*C_b_*, liquid bulk concentration (g/L);

*C_MLSS_*, mixed liquor suspended solids concentration (g/L);

*E*, activation energy and typically 9.217 (kJ·mol^−1^);

*f’*, fractional amount of total foulants contributing to deposit growth (−);

*g_o_*, adjustable parameter or cake removal factor (−);

*J*_0_, initial filtrate flux of clean membrane (m·s^−1^);

*J_u_*, unblocked flux (m·s^−1^);

*k_Ab_*, area constant parameter for blocked pores (−);

*k*_ω_, angular velocity factor (−);

*m*, flow consistency index (Pa·s ^n^);

*n*, flow behaviour index (−);

*PT*, transmembrane pressure at membrane periphery (Pa);

*Q*, volumetric flow rate (m^3^·s^−1^);

*Q_b_*, blocked volumetric flow rate (m^3^·s^−1^);

*Q*_0_, initial volumetric flow rate (m^3^·s^−1^);

*Q_u_*, unblocked volumetric flow rate (m^3^·s^−1^);

*R’*, specific protein layer or cake layer resistance (m/kg);

*R_b_*, resistance of solids deposit over a region of membrane (m^−1^);

*R_bo_*, initial resistance of solids deposit (m^−1^);

*R_c_* = *R’_c_ θ_c_*, total net cake’s resistance (m^−1^);

*R’_c_*, specific cake resistance (m^−2^);

*R_eNN_*, radial Reynolds number (−);

*R_g_*, universal gas constant 8.3145 × 10^−3^ (kJ·K^−1^·mol^−1^);

*r_i_*, membrane’s inner radius (m);

*R_in,b_*, membrane’s resistance & resistance from pore constriction (m^−1^);

*r_o_*, membrane’s outer radius (m);

*r’_0_*, distance radius from the spinning axis (m), thus (*r’_0_ = r_o_ − r_i_*);

*R_m_*, clean membrane’s resistance (m^−1^);

*r_p_*, radius of membrane pore (m);

*t*, filtration time (s);

*t_b_*, time at which a membrane region was first blocked (s);

TMP, transmembrane pressure (Pa);

*T_room_*, room temperature (°C);

α, pore blockage parameter (m^2^/kg);

α_v_, air scouring coefficient (−);

β, pore constriction parameter (kg);

$\dot{\gamma }$, shear rate (s^−1^);

δ, resistance distribution constant (m^−1^);

△P, cake’s transmembrane pressure (Pa);

δ_m_, membrane thickness (m);

θ_c_, cake’s depth or thickness (m);

µ, viscosity (Pa·s);

υ, fluid kinematic viscosity (m^2^·s^−1^);

ρ_f_, fluid’s density (kg·m^−3^);

σ_a_, adjustable parameter related to pore constriction (m^3^·kg);

τ, cake water content (−);

ω, angular velocity (rad·s^−1^).
